# Household Disposal of Pharmaceuticals as a Pathway for Aquatic Contamination in the United Kingdom

**DOI:** 10.1289/ehp.8315

**Published:** 2005-08-09

**Authors:** Jonathan P. Bound, Nikolaos Voulvoulis

**Affiliations:** Centre for Environmental Policy, Imperial College London, London, United Kingdom

**Keywords:** drugs, prescriptions, risk assessment, survey, wastewater treatment

## Abstract

Pharmaceuticals are produced and used in increasingly large volumes every year. With this growth comes concern about the fate and effects of these compounds in the environment. The discovery of pharmaceuticals in the aquatic environment has stimulated research in the last decade. A wide range of pharmaceuticals has been found in fresh and marine waters, and it has recently been shown that even in small quantities, some of these compounds have the potential to cause harm to aquatic life. The primary pathway into the environment is the use and disposal of medicines; although much of the research in the area currently focuses on the removal of pharmaceuticals during sewage treatment processes, disposal via household waste might be a significant pathway requiring further research. To investigate the household disposal of unused and expired pharmaceuticals as a source of pharmaceutical compounds in the environment, we carried out a survey and interviewed members of 400 households, predominantly from southeastern England. We used the information on when and how they disposed of unfinished pharmaceuticals to construct a conceptual model to assess the pathways of human pharmaceuticals into the environment. The model demonstrated that disposal of unused pharmaceuticals, either by household waste or via the sink or toilet, may be a prominent route that requires greater attention.

The presence and potential adverse effects of pharmaceuticals in the aquatic environment have begun to receive increasing attention in the popular and scientific press in recent years. This increase is largely a result of a number of scientific papers published in the 1990s that reported trace levels of pharmaceuticals detected in environmental samples, including sewage effluent, surface water, groundwater, and even drinking water, mainly in European countries (Buser et al. 1998; Daughton and Ternes 1999; Halling-Sorensen et al. 1998; Heberer 2002; Jones et al. 2001; Kolpin et al. 2002; Ternes 1998; Ternes et al. 1999). The existence of pharmaceuticals in the U.K. aquatic environment has been established, but the extent of their distribution and the possible ecotoxicologic consequences associated with their presence are less clear. Pharmaceuticals are produced and used in very large volumes, and their use and diversity are increasing every year. Estimates based on the number of prescriptions issued suggest that around 100 tons of drugs were prescribed in Germany in 1995 (Ternes 1998). In the United Kingdom in 2000, use exceeded 10 tons/year for each of the top 25 compounds, and the amount of the top three compounds prescribed [acetaminophen (paracetamol), metformin hydrochloride, and ibuprofen] was > 100 tons/year each (Jones et al. 2002). Recent research has shown that these compounds could have a negative effect on the aquatic environment (Jones et al. 2003). Observed environmental effects are limited mainly to the feminizing activity of endocrine-disrupting compounds such as the synthetic hormone 17α-ethinyl estradiol on fish near wastewater treatment works (WWTW) outfalls (Jobling et al. 1998; Länge et al. 2001; Routledge et al. 1998). Other concerns include the development of antibacterial resistance either in or near WWTWs (Schwartz et al. 2002) or in the environment as a consequence of veterinary drug use (Petersen et al. 2002). Detection of these negative effects in the environment is difficult; although *in vivo* and *in vitro* laboratory tests generally show that the toxic effects of these compounds are not seen at the low levels currently detected in the environment, the possibility of variations in sensitivity, chronic exposure, and mixture effects such as concentration addition and synergism (Altenburger et al. 2004; Cleuvers 2004; Richards et al. 2004) mean that other negative effects cannot be ruled out. As a result, risk assessment guidance has been developed to predict the environmental impact caused by new pharmaceuticals [Bound and Voulvoulis 2004; European Agency for the Evaluation of Medicinal Products (EMEA) 2005; U.S. Food and Drug Administration (FDA) 1998].

There are two main routes for pharmaceuticals to enter the environment. The first is via the effluent from WWTWs after excretion from the body (Figure 1). After administration, a significant proportion of a pharmaceutical may pass through the body unmetabolized. The degree to which a compound is changed in the body depends on its structure and mechanism of action. The β-blocker nadolol may pass through the human body completely unmodified (RxList 2005a). In contrast, only 3% of the parent form of the antiepileptic carbamazepine is excreted unchanged in the urine (RxList 2005b); the rest may be conjugated or hydroxylated and also released in the feces. Release via this pathway is governed by the pharmacology of the drug and the efficiency of the WWTW. Excretion rates of many pharmaceuticals, such as those shown in Table 1, can be found in both medical (Martindale 1993) and environmental literature (Calamari et al. 2003; Jjemba, in press). The exact rates also depend on the dosage and the physiology of the individual.

Data on WWTW removal efficiencies are sparse and are largely dependent on the facilities at individual WWTWs and on variables such as local rainfall and temperature (Table 2). For example, only 9% of diclofenac was found to be removed by biologic filtration, whereas 75% was removed by activated sludge treatment (Stumpf etal. 1999). Because these data are required by the draft European Union guidelines on risk assessment (EMEA 2005), there will presumably be an increase in research in this area.

The second route by which pharmaceuticals can enter the environment is by the disposal of out-of-date or unwanted medicines, which may occur via the sink/toilet or in household waste that is then taken to landfill sites (Figure 1). Entry into the environment by this route is dependent on the habits of the patient and the efficiency of prescription practices leading to fewer unfinished prescriptions. Discarded pharmaceuticals are defined in the United Kingdom by the Controlled Waste Regulations 1992 [Her Majesty’s Stationery Office (HMSO) 1992] as clinical waste and as such are controlled by the Special Waste Regulations 1996 (HMSO 1996). According to this legislation, such waste may be disposed of in landfill sites designed to accommodate hazardous waste, or it may be incinerated. However, once dispensed to or purchased by a member of the public, any unwanted pharmaceutical products are classified as household waste, and their disposal is not subject to any controls. Manufacturer packaging usually recommends disposal by returning to the pharmacist; however, disposal via the sink/toilet or in normal household waste is common. Pharmaceuticals in landfill sites are subject to biologic degradation processes, but some may persist and even leach into surrounding groundwater and rivers (Ahel etal. 1998; Holm etal. 1995; Schwarzbauer etal. 2002).

An investigation into the disposal habits of the American public found that only 1.4% of the people they surveyed returned unused medication to the pharmacy, whereas 54% threw them away and 35.4% disposed of them in the sink/toilet (Kuspis and Krenzelok 1996). These methods of disposal result from U.S. Drug Enforcement Administration regulations that strictly control the transfer of drugs and controlled substances. It is possible for some institutions to return unwanted drugs via organizations affiliated with the Returns Industry Association, a group of licensed “reverse distributors” that offer a return and disposal service (Daughton 2003b). Although regulations vary among U.S. states, most pharmacies cannot accept returns from patients. Measures to allow the return of unused medication from long-term care facilities have been passed or are being considered in some states. Developments in legislation are listed on the National Conference of State Legislatures website (NCSL 2005). Some states will also allow the redistribution of drugs within their expiration date, although they do not permit the return of drugs by private individuals. This service is therefore limited largely to medicines that never leave pharmacies and care facilities.

A source of concern is that, at the pharmacies questioned, 68% of unreturnable medicines were disposed of in nonhazardous waste or via the drain. Traditionally, disposal advice to consumers has been limited to flushing down the toilet or, in some cases, burning or grinding and discarding in household waste (Pray and Pray 2004), but emerging environmental concerns mean that this is sometimes modified (Daughton 2003b). People are first advised to check whether local pharmacies or doctors are able to receive returns or whether hazardous waste facilities exist in the area. As a last resort, disposal in household waste is deemed to be less harmful than disposal via the sewage system (Boehringer 2004). A study by Braybrook et al. (1999), designed to examine ways to streamline the prescription process in order to reduce costs, looked at some of the reasons people gave for returning unused pharmaceuticals to the pharmacy. The most common reason was a change of medication. Most items (80%) were returned within a year of their prescription date, but some people returned the medicines only after the infrequent removal of unwanted items that have built up over time, with some products being returned 13 years after they were dispensed.

The aim of the present study was to identify and assess the significance of the different pathways of pharmaceuticals from the household to the environment. Knowledge of the motivation behind different disposal methods is useful in the management of the release of pharmaceuticals in the environment and in the assessment of the associated risk. This project aimed to demonstrate the possible importance of household disposal of unused medicines as a pathway into the aquatic environment.

## Materials and Methods

Pharmaceuticals are a large and varied class of compounds with diverse properties and applications. To facilitate their study, they are often grouped by their therapeutic action. We targeted eight therapeutic groups in this study. We used various criteria, including volume of prescription, toxicity, and evidence of presence in the environment, in the selection process. Table 3 presents a summary of the factors that cause concern (risk indicators), with examples of pharmaceuticals within the groups.

A survey was devised to investigate disposal patterns of the eight selected groups of pharmaceuticals. This survey was part of a study into the disposal of household hazardous waste supported by the Environment Agency of England and Wales (Slack et al. 2005b). Respondents were asked whether they ever had any of the types of medicines and when and how they disposed of them. Information about the age, sex, education, profession, and postal code of the respondent in order to assess socioeconomic status was collected. Respondents gave their written informed consent to this information being used anonymously in our study. Only closed-ended questions were used, with the questioner specifying possible answers. These questions have the advantages of being quick to administer, easy to answer, and easier to analyze and interpret than are open-ended questions (Petersen 2000). Where list questions were employed, no limit was placed on the number of answers that could be given, so that respondents were not required to choose a single answer when it did not wholly represent their attitude or behavior.

Using Equation 1 and the method of McCall (1982), we calculated that the number of respondents required to obtain a representative sample, *n*, was 384. We divided the total population into four groups: those who lived in population centers of ≥250,000 (cities), 249,999–50,000 (very large towns), 49,999–10,000 (mid-sized towns), and < 9,999 (small towns/villages). The numbers of people estimated to live in each type of area were extrapolated down and applied to the sample size in order to achieve a representative spread:


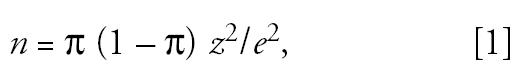


where *n* is the estimated sample size required for desired precision and confidence, 384; πis the preliminary estimate of proportion opposed to this initiative within the population, 0.5; *z* is the two-tailed value of the standardized normal deviation associated with the desired level of confidence; and *e* is the desired precision, half the maximum acceptable confidence interval, here 0.05.

**Figure 1:**
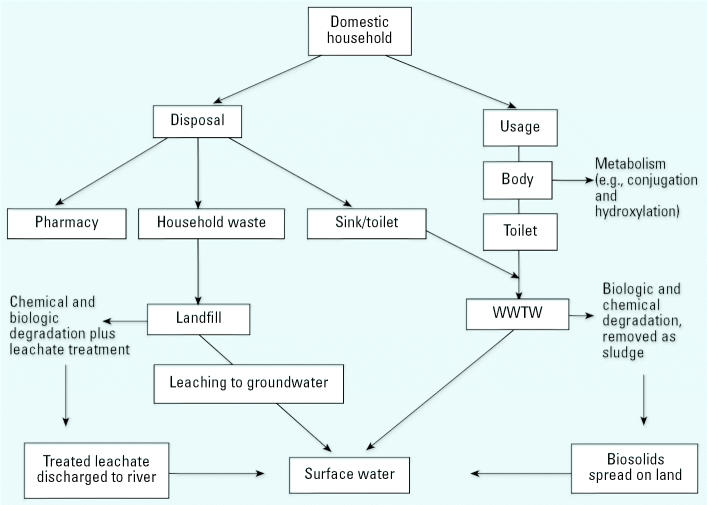
Pathways of drug fate from domestic households to the environment.

We used a model based on the flow diagram in Figure 1 to quantify the amount of pharmaceuticals that reach the environment by the various pathways shown. The division between the use and disposal of drugs is based on responses from subjects who said they finished the prescription and therefore had nothing to dispose of and those who said they disposed of drugs at another time (e.g., when the drug expired). Because it was difficult to collect information on the proportion of these medicines that were used before disposal, a number of assumptions had to be made. The main assumption was that subjects who said that they had some medicine to dispose of first consumed 50% of the prescription, disposing of the remaining 50%. We also assumed that all prescriptions of each individual drug contained the same quantity and strength of drugs. These assumptions limit the accuracy of the present model. The most reliable way to establish the proportion of prescriptions that remain unfinished and the method of disposal chosen would be to collect unwanted medicines directly from households.

As with any survey, the quality of the results depends on on the truthfulness of the responses. Forgetfulness and embarrassment about socially stigmatized medication, for example, may lead to misreporting and incorrect estimates. People may feel pressured to give the answers that they think are the “right” ones, those that are more socially acceptable, or those that they believe the questioner wants to hear. This was minimized by the passive questioning style, with as little prompting as possible. In a review of the accuracy of patient self-reporting, Evans and Crawford (1999) found mixed results: patient recall, as one might expect, was more reliable over short time periods and less so in elderly patients.

We did not request specific data about types and amounts of medication, so the reported data should be less affected by these problems. If such information was required, a patient diary for recording all incidences of medication would be the most effective way of obtaining it. The answers relating to behavior are less dependent on recall and more dependent on opinion and personal preference. However, a patient diary would also be useful in charting the methods of disposal and the exact volumes concerned. This project has served as a pilot study, establishing the need for more specific data, and a more detailed drug collection study is now under way to provide accurate information to supplement the proposed model.

The model uses data that describe the percentage of the parent compound that passes through the body unchanged. It is possible that conjugates could be hydrolyzed back to the parent compound in the environment. It is also possible that metabolic products could be more toxic in the environment than the parent compound. Where such data are available, they could be incorporated. They were not, however, incorporated in the model presented here.

## Results

**Figure 2:**
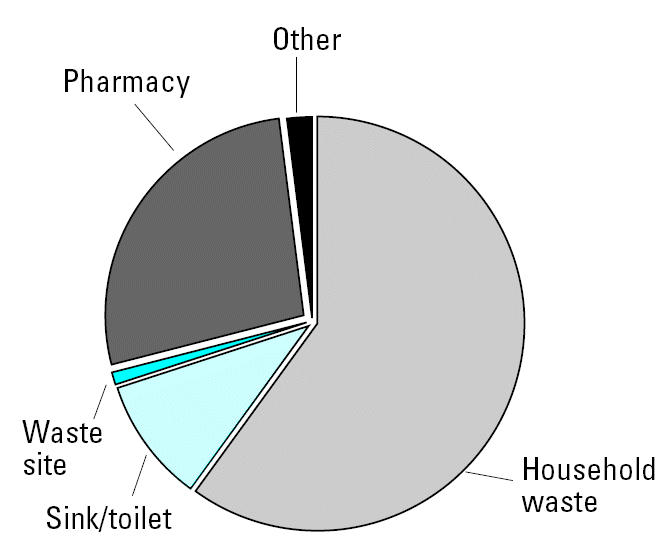
Subjects’ usual disposal methods for pharmaceuticals.

The survey was carried out in southeastern England during the summer of 2003. The minimum sample size was exceeded with 392 people interviewed (54.8% female, 45.2% male), closely reflecting the actual distribution in the United Kingdom (51.3% female, 48.7% male). The subjects were also spread evenly across age ranges and family types. Almost everyone (98%) had some type of pharmaceutical in their house; most (60.2%) had a mixture of over-the-counter (OTC) and prescription medicines, whereas 30.7% had only OTC medicines and 9.1% had only prescription medicines. Responses indicate that just more than half (52.8%) finish their medication and hence have none to dispose of. Around a third (30.7%) keep them until the expiration date, and 12.2% dispose of them when the treatment has been completed. Figure 2 describes the disposal of unwanted pharmaceuticals. Two-thirds (63.2%) discard them in household waste, with the remainder returning them to a pharmacist (21.8%) or emptying them into the sink or toilet (11.5%). A small number took them to municipal waste sites that sometimes have special waste facilities.

The data can be broken down into the eight selected pharmaceutical groups to show how behavior varies with respect to drug type (Table 4). Nearly 80% of people consume all of the painkillers that they buy or are prescribed, whereas the figure for antibiotics (18%) is worryingly low. Household waste was the most popular disposal method for all types of drugs. Although the average rate of sink/toilet disposal for all drug types is 11.5%, none of the 90 people who had hormone treatments admitted to flushing them down the toilet, with the number returning them to the pharmacy increasing accordingly.

**Figure 3:**
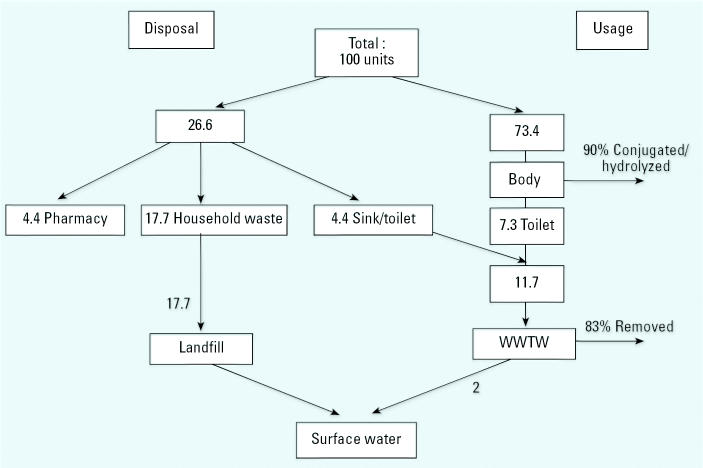
The fate of metoprolol by units used.

The information on the disposal of two different types of pharmaceuticals, metoprolol and ibuprofen, along with figures on the elimination of the compound in the human body and WWTW removal efficiencies, was used to model the relative importance of the pathways into the environment. Metoprolol succinate is a β-blocker, mainly used in the treatment of high blood pressure. It is available only by prescription in the United Kingdom. Figure 3 is a mass balance flow chart showing the fate of 100 units of the parent compound. Only 46.8% of respondents who had been prescribed β-blockers said that they finished the prescription. Assuming, as previously stated, that those people took half of the medication, then 26.6 units are disposed of and 73.4 units of the active ingredient are consumed. Because 90% of the medication taken is modified by the body, this leaves 7.3 units of active ingredient that are introduced to the wastewater system (Huschek et al. 2004). When combined with the 4.4 units (16.7%) of drugs that are put down the drain, this results in a total of 11.7 units entering WWTWs. Here, 83% is removed (Ternes 1998), leaving 2 units to be discharged into surface water. Of the 26.6 units that are unused, 4.4 are returned to the pharmacy whereas 17.7 units, nearly 10 times as much as is released into the environment from WWTWs, are put into household waste that is subsequently taken to a landfill. Once there, some will be removed by biologic and chemical degradation within the landfill, some will be collected at leachate treatment plants and subjected to similar processes as in the WWTWs and then released into surface water, and some may leach directly into the surrounding groundwater and possibly rivers.

In the case of metoprolol, household disposal might be a significant pathway into the environment. This is because the drug is not removed or modified by the body, nor is it modified by WWTW processes. The literature currently reflects a bias toward research of WWTW treatment rather than landfill leachate that may not fully address the risks of pharmaceuticals to the environment. It is also important to note that the sludge generated during WWTW treatment may be itself land-filled or spread on agricultural land—the risk of pharmaceuticals is not necessarily removed, just moved. Millions of tons of sewage sludge are generated in the European Union every year. The proportion of the pharmaceutical load contained within the solid waste products of WWTWs depends largely on the properties of the drug, especially the octanol–water coefficient (*K*_OW_), which is an indicator of the likelihood that the compound will be partitioned into the solid phase. Other important interactions are the sorption to organic matter, surface adsorption to mineral constituents, ion exchange, complex formation with metal ions such as Ca^2+^, Mg^2+^, Fe^3+^, or Al^3+^, and hydrogen bonding (Diaz-Cruz et al. 2003). Once these “biosolids” have been spread on agricultural land or landfilled, degradation may continue, but there is also the potential for soil and groundwater contamination, runoff, and even adverse effects on plants or animals reared on the land (Jjemba 2002; Xia etal. 2005).

**Figure 4:**
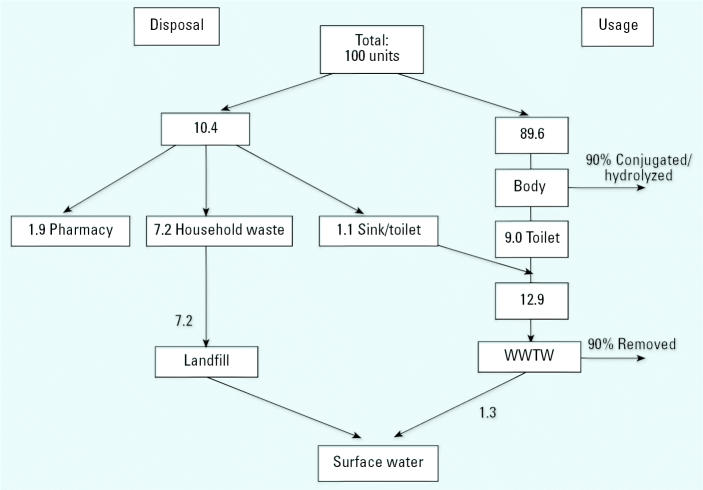
The fate of ibuprofen by units used.

The same model applied to ibuprofen (Figure 4) shows that usage is a more prominent pathway than it is for metoprolol. Results of the survey showed that fewer people (20.8%) had any painkillers to dispose of. Assuming they consumed half of these, only 10.4 units require disposal. Therefore, even though from the model the rates of elimination in the body and WWTWs are comparable with those of metoprolol, the ratio of the active ingredient entering landfill sites compared with that entering surface water from WWTWs is 5.5:1 for ibuprofen (the ratio for metoprolol is 8.9:1). This demonstrates that both human behavior and pharmacologic properties of the active ingredient are important in assessing the significance of the different pathways into the environment.

## Discussion

Despite advice on pharmaceutical packaging that recommends the return of unused medicines to pharmacies, or occasionally to flush them down the toilet, the predominant method of disposal in the United Kingdom was found to be via household waste. Although this result is similar to that found in the United States by Kuspis and Krenzelok (1996), the figures for those returning their unused medication to the pharmacy (21.8% in the United Kingdom compared with 1.4% in the United States) and those who disposed of the medicines down the toilet (11.5% in the United Kingdom and 35.4% in the United States) may reflect the disparity between regulations and advice in the two regions. The answers given to the survey conducted in the present study suggest that there may be a significant quantity of pharmaceuticals entering the household waste stream in the United Kingdom. This is of potential concern because medicines deposited in their original form in landfill sites bypass the human body and WWTWs. It is therefore possible that even though comparatively small quantities may travel by this pathway, it could have increased significance because of this avoidance of removal mechanisms. The variation in these removal rates makes it difficult to generalize the relevance of the different pathways into the environment for all medicines.

The model described in Figures 3 and 4 is intended to show that the household disposal of medicines is worthy of consideration in the risk assessment and management process. In its current form, this model is not capable of predicting the precise amounts of pharmaceuticals entering the environment by each pathway. However, with the limited information available, it does show that, under the conditions proposed, the disposal pathway is a potential cause for concern and should figure more prominently in the investigations into the presence of pharmaceuticals in the aquatic environment. The model also shows how different drugs will favor different pathways. More than twice the percentage of people questioned said they disposed of β-blockers compared with painkillers. This could be due to changing prescriptions or the fact that people foresee a future use for painkillers. It could also be related to the patient’s perception of risk about the relative dangers of the two types of drugs (Bound et al., in press).

With other compounds, the dominant factor could be the metabolism or the stability within the WWTWs. The model gives figures for the proportion of the parent compound that passes through the body and the WWTWs unchanged. Some of the other products of these processes may also have ecotoxicologic properties. It may be possible to modify the model where further knowledge about the pharmaceutical is available. In the case of metoprolol, Huschek et al. (2004) found that some of the other metabolic products also showed pharmaceutical activity. If these are included in the calculation, a total of 34% of the drug was excreted in active forms. Where this information is known, it could also be included in the calculation. However, “active” refers to the pharmacologic properties that may not necessarily coincide with environmental toxicology.

The most straightforward way to eliminate the risk posed by the disposal pathway would be to reduce the quantity of drugs being improperly discarded. One possibility is to increase the prominence of product labeling and the provision of stronger advice on how to dispose of any remaining drugs. The results of the survey showed that, although half of people finished their prescriptions, reasons for disposal included expiration (30.7%) and completion of treatment before finishing the prescription (12.2%). This is understandable for OTC medications. However, in the case of prescription medication, this indicates that the instructions that accompany the prescription have not been adhered to, because completion of the treatment and the end of the prescription should coincide if normal practice is followed. This level of noncompliance (patient not following completely the instructions from their physician) is similar to estimates elsewhere (Donovan and Blake 1992).

Patients may deviate from recommendations for many reasons: they may be avoiding unpleasant side effects; they may believe that, because symptoms have been alleviated, there is no need to continue medication; or it could simply be forgetfulness. A possible solution would be to increase the information given to patients by doctors and pharmacists about the need to complete courses of medication and the importance of safe disposal when medicines remain unused. However, if up to 50% of patients do not follow advice that could have important impacts on their own health, will they be prepared to alter their behavior based on environmental concerns? A Canadian survey reported that, although > 50% of people said that they read the labels of OTC medications, only 2% said that they read product packaging to discover appropriate disposal methods. However, when directly prompted about disposal information, 57% stated that they did (COMPAS Inc. 2002). These factors would seem to undermine the efficacy of product labeling as a means of reducing improper disposal.

Investigations into environmental contamination via landfill leachate (Ahel et al. 1998; Eckel et al. 1993; Holm et al. 1995; Schwarzbauer et al. 2002; Slack et al. 2005a) are far less common than similar studies into pollution from WWTWs. They are also often concerned with sites that have received large quantities of pharmaceuticals in bulk as part of industrial disposal rather than just household waste. Modern landfill sites are usually equipped with linings capable of preventing a high proportion of leachate from escaping into the surrounding groundwater. Where this is the case, leachate treatment plants are often employed to reduce or remove harmful contaminants before their release into surface waters. These facilities are potentially capable of intensive waste management systems partly because of the low volumes involved compared with WWTWs or drinking-water plants. Such processes include ozonation, nanofiltration, and activated carbon adsorption (Wintgens et al. 2003; Wu et al. 2004). Advanced facilities such as these are not currently widespread, but they could be introduced to reduce the release of pharmaceuticals, pesticides, and endocrine disruptors into the aquatic environment. Older sites and those in developing countries are unlikely to have modern membrane liners to prevent leaching, although some may rely on natural geologic features to minimize groundwater contamination. Further studies on the concentrations of pharmaceutical compounds within landfill sites and in leachate would be informative, and if necessary, those sites not equipped with the necessary treatment facilities could be upgraded.

Current and proposed risk assessment guidelines in the European Union (EMEA 2005) and the United States (FDA 1998) do not consider the disposal pathway when calculating the predicted environmental concentrations of medicines. Applicants for new licenses could use studies such as the one presented here to predict the proportion of medicine that will be disposed of in general waste using figures for local prescription practices and public behavior. This approach may be considered too time-consuming when compared, for example, to earlier “worst-case scenario” approaches (EMEA 2001) that assume that all of the prescribed drug will end up in the surface water. But the more recent revisions account for removal in the body and WWTWs. This optimization of the process means that some compounds that might have been recommended for assessment under the earlier system will now be shown to be sufficiently safe because a high proportion of the compound is degraded to a less toxic form. However, if a significant proportion of the drug is not undergoing the transformation within the patient and WWTWs, there is a possibility that enough of the active ingredient would reach the environment to trigger further investigation. We believe that a complete risk assessment framework should give some consideration to the disposal pathway.

Daughton (2003a) advocated the development of a database to catalogue the distribution of prescription and OTC drugs (information on the latter in particular is difficult to obtain at the present). Regional variations in the supply of pharmaceuticals could be coupled with data on the metabolic breakdown and WWTW degradation (this could be locally optimized to include the type of treatment processes in use in a specific region, e.g., primary, secondary, activated sludge) to more accurately predict the release of a pharmaceutical in the environment. Furthermore, this information could be combined with data on the disposal of unused medicines, as proposed in this study. Where facilities exist, information on returns from pharmacies and hospitals could also be incorporated to provide a more effective method for the prediction of environmental concentrations. Knowledge about the presence of drugs in household waste could benefit the management of risks to the environment. Minimizing the disposal pathway could be more effective and less costly than extensive WWTW modifications or other remediation steps.

## Figures and Tables

**Table 1:** Urinary excretion rates of unchanged active ingredient for selected pharmaceuticals.

Drug	Therapeutic class	Parent compound excreted (%)	Reference
Ibuprofen	Painkiller	10	Dollery 1991
Paracetamol	Painkiller	4	Huschek et al. 2004
Amoxycillin	Antibacterial	60	Martindale 1993
Erythromycin	Antibacterial	25	Huschek et al. 2004
Sulfamethoxazole	Antibacterial	15	Hirsch et al. 1999
Atenolol	β -Blocker	90	Dollery 1991
Metoprolol	β -Blocker	10	Huschek et al. 2004
Carbamazepine	Antiepileptic	3	Huschek et al. 2004
Felbamate	Antiepileptic	40–50	RxList 2005c
Cetirizine	Antihistamine	50	RxList 2005d
Bezafibrate	Lipid regulator	50	Ternes 1998

**Table 2:** Removal of selected pharmaceuticals in WWTWs.

Drug	Percent WWTW removal	Treatment process	Reference
Bezafibrate	99.5	Activated sludge	Kreuzinger et al. 2004
	83	Activated sludge	Ternes 1998
	50	Activated sludge	Stumpf et al. 1999
	27	Biologic filter	Stumpf et al. 1999
Carbamazepine	10	Activated sludge	Kreuzinger et al. 2004
	7	Activated sludge	Ternes 1998
Diclofenac	75	Activated sludge	Stumpf et al. 1999
	69	Activated sludge	Ternes 1998
	9	Biologic filter	Stumpf et al. 1999
	0	Average of 7 WWTWs	Lee et al. 2003
17α -Ethinyl estradiol	78	Activated sludge	Ternes et al. 1999
	64	Biologic filter	Ternes et al. 1999
Gemfibrozil	69	Activated sludge	Ternes 1998
	46	Activated sludge	Stumpf et al. 1999
	16	Biologic filter	Stumpf et al. 1999
	5	Average of 7 WWTWs	Lee et al. 2003
Ibuprofen	99	Activated sludge	Kreuzinger et al. 2004
	90	Activated sludge	Ternes 1998
	87	Average of 7 WWTWs	Lee et al. 2003
	80–100	Activated sludge	Kanda et al. 2003
	75	Activated sludge	Stumpf et al. 1999
	60–70	Activated sludge	Carballa et al. 2004
	65	Biologic filter	Rodriguez et al. 2003
	22	Biologic filter	Stumpf et al. 1999
	14–44	Biologic filter	Kanda et al. 2003
Indomethacin	40	Average of 7 WWTWs	Lee et al. 2003
Ketoprofen	69	Activated sludge	Stumpf et al. 1999
	48	Biologic filter	Stumpf et al. 1999
	18	Average of 7 WWTWs	Lee et al. 2003
Metoprolol	83	Activated sludge	Ternes 1998
Naproxen	78	Activated sludge	Stumpf et al. 1999
	70	Average of 7 WWTWs	Lee et al. 2003
	66	Activated sludge	Ternes 1998
	40–55	Activated sludge	Carballa et al. 2004
	45	Biologic filter	Rodriguez et al. 2003
	15	Biologic filter	Stumpf et al. 1999
Propranolol	96	Activated sludge	Ternes 1998
Sulfamethoxazole	67	Activated sludge	Carballa et al. 2004

**Table 3:** Selected pharmaceutical groups and their environmental risk indicators.

Drug	Examples	Risk indicator	References
Painkillers	NSAIDS (e.g., ibuprofen), other analgesics (e.g., acetaminophen)	Very high prescription and OTC volumes; detected in the environment	Buser et al. 1999
Antibiotics	Penicillins, sulfamethoxazole	High volumes; detected in the environment; concerns over toxicity and antibacterial resistance	Berger et al. 1986 Hirsch et al. 1999 Leff et al. 1993 Wollenberger et al. 2000
β -Blockers	Propranolol, metoprolol	High volumes; detected in the environment	Calamari et al. 2003 Ternes 1998
Antiepileptics	Carbamazepine, phenobarbital	High volumes; long-term prescriptions; persistent	Andreozzi et al. 2002
Lipid regulators	Statins (e.g., atorvastatin), clofibrate	Long-term prescriptions; commonly detected	Buser et al. 1998 Heberer et al. 1997
Antidepressants	Fluoxetins, risperidone	Subject of toxicity testing	Brooks et al. 2003
Hormone treatments	Contraceptive pills, 17α -ethinyl estradiol, hormone replacement	Most extensively studied toxicologic properties; widely detected	Arcand-Hoy et al. 1998 Länge et al. 2001 Purdom et al. 1994 Rodgers-Gray et al. 2000
Antihistamines	Loratadine, cetirizine	Commonly held nonprescription medicine	

Abbreviations: NSAIDS, nonsteroidal anti-inflammatory drugs; OTC, over-the-counter.

**Table 4:** Disposal characteristics (%) based on drug type.

		When				How			
Drug	Present	Empty	Expired	Treatment finished	Other	Trash bin	Sink/toilet	Pharmacy	Other
Painkiller	94.1	79.2	18.4	2	0.4	69.6	10.9	18.5	1
Antihistamine	45.9	61.4	33	3.7	1.9	75.3	9.1	14.3	1.3
Antibiotic	56.4	17.6	10.5	69.9	2.1	71.4	3.6	14.3	10.7
Antiepileptic	2	66.7	22.2	11.1	0	100	0	0	0
β -Blocker	11.2	46.8	12.8	38.3	2.1	66.7	16.7	16.7	0
Hormone	23.2	68.1	4.3	26.6	1.1	75	0	25	0
Lipid regulator	6.9	41.4	6.9	51.7	0	66.7	0	0	33.3
Antidepressant	9.7	53.7	14.6	29.3	2.4	66.7	0	33.3	0
